# Artificial intelligence abstracts from the European Congress of Radiology: analysis of topics and compliance with the STARD for abstracts checklist

**DOI:** 10.1186/s13244-020-00866-7

**Published:** 2020-04-25

**Authors:** Thomas Dratsch, Liliana Caldeira, David Maintz, Daniel Pinto dos Santos

**Affiliations:** Institute of Diagnostic and Interventional Radiology, University Hospital of Cologne, Kerpener Str. 62, 50937 Cologne, Germany

**Keywords:** Machine learning, Artificial intelligence, Radiology, Congresses as topic, Data reporting

## Abstract

**Objectives:**

To analyze all artificial intelligence abstracts presented at the European Congress of Radiology (ECR) 2019 with regard to their topics and their adherence to the Standards for Reporting Diagnostic accuracy studies (STARD) checklist.

**Methods:**

A total of 184 abstracts were analyzed with regard to adherence to the STARD criteria for abstracts as well as the reported modality, body region, pathology, and use cases.

**Results:**

Major topics of artificial intelligence abstracts were classification tasks in the abdomen, chest, and brain with CT being the most commonly used modality. Out of the 10 STARD for abstract criteria analyzed in the present study, on average, 5.32 (SD = 1.38) were reported by the 184 abstracts. Specifically, the highest adherence with STARD for abstracts was found for general interpretation of results of abstracts (100.0%, 184 of 184), clear study objectives (99.5%, 183 of 184), and estimates of diagnostic accuracy (96.2%, 177 of 184). The lowest STARD adherence was found for eligibility criteria for participants (9.2%, 17 of 184), type of study series (13.6%, 25 of 184), and implications for practice (20.7%, 44 of 184). There was no significant difference in the number of reported STARD criteria between abstracts accepted for oral presentation (*M* = 5.35, SD = 1.31) and abstracts accepted for the electronic poster session (*M* = 5.39, SD = 1.45) (*p* = .86).

**Conclusions:**

The adherence with STARD for abstract was low, indicating that providing authors with the related checklist may increase the quality of abstracts.

## Key points

- Classification tasks and CT imaging were the most common topics of ECR artificial intelligence abstracts.
- The adherence with the STARD for abstracts checklist was in general low.
- Adherence for these essential STARD checklist elements was not higher among abstracts accepted for oral presentation compared to abstracts accepted for the electronic poster session

## Introduction

New machine learning techniques, especially deep neural networks, hold the promise to revolutionize many aspects of radiology and have gained immense public and professional attention over the last few years [1, 2]. This has led to a sharp increase in publications [3–7], the founding of new journals [8], and FDA approval for new diagnostic algorithms [9–11]. This rapid expansion of the field is also reflected by the introduction of machine learning and artificial intelligence as a new topic at the 2019 European Congress of Radiology. With this increased scientific output, it may be helpful to take a step back and try to get a bigger picture of the research landscape. Does this increased scientific output consist of quality research with a strong methodology or are machine learning techniques applied without considering potential pitfalls to quickly produce papers that follow the latest trend?

Because conference abstracts can be a preview of future publications, analyzing them can give insights into where the field is headed: What are the problems that machine learning models are mainly used to solve? What are the pathologies with the most research focus? And what body regions and modalities get the most attention? All these questions can be answered by taking a closer look at the abstracts from the European Congress of Radiology (ECR) 2019. Besides the thematic focus of the abstracts, we also assessed the adherence of abstracts to established reporting guidelines. As previous efforts have shown, a large proportion of studies focusing on the evaluation of AI algorithms are lacking important features, such as external validation, that ensure robust performance in a clinical setting [12]. Reporting guidelines, such as STARD [13], CONSORT [14], STROBE [15], and PRISMA [16], can help when conceptualizing a study and during the publication process to ensure that all necessary information is included in the final paper. For the purpose of this investigation, we compared submitted abstracts to the STARD criteria for abstracts [13]. The goal of the STARD initiative is to improve the way studies of diagnostic accuracy are reported by ensuring that all relevant information to sufficiently judge and reproduce a given study is included in the final paper. We chose the STARD criteria for this study for two reasons: first, the STARD criteria were specifically developed to assess studies of diagnostic accuracy. Because one of the most common applications of machine learning techniques in medicine is the development of new diagnostic algorithms, the STARD criteria are a good fit to evaluate this particular kind of study. Second, the STARD for abstracts checklist was specifically developed for conference abstracts and therefore well suited for the task of analyzing the abstracts of the ECR 2019.

To sum up, the goal of the present study was to analyze all artificial intelligence abstracts presented at the European Congress of Radiology (ECR) 2019 with regard to their topics and their adherence to the Standards for Reporting Diagnostic accuracy studies (STARD) checklist.

## Materials and methods

### Abstracts

ECR 2019’s respective websites (EPOS and ECR online) and the book of abstracts were analyzed by one of the authors to identify all abstracts that were submitted under the topic “AI,” which resulted in a final sample of 184 abstracts that were used as the basis for all further analyses.

In addition to the title and text, we also retrieved the country of origin. Abstracts were divided into two main categories: oral presentation (scientific presentation and MyT3: My Thesis in 3 Minutes) and electronic poster session.

### STARD criteria

For the purpose of this study, we used the STARD criteria for abstracts [13]. The criteria are organized according to the general structure of a scientific paper (Background, Methods, Results, and Discussion) and focus on the information crucial to replicable and criticizable research. See Table 1 for an overview of the eleven STARD criteria for abstracts. The 11th criterion—registration number—was not used in the present study because registration number and name of registry are usually not required for abstracts submitted to the ECR.

**Table 1:** STARD criteria for abstracts (adapted from Cohen et al. [13])

Category	Item
**Study of diagnostic accuracy**	Study uses at least one measure of diagnostic accuracy (e.g., sensitivity, specificity, predictive values, or AUC)
**Study objectives**	Objectives of the study are clearly stated
**Data collection**	Provides information on whether the study was prospective or retrospective
**Eligibility criteria**	Eligibility criteria for participants and settings where the data were collected
**Type of series**	Consecutive, random, or convenience series
**Index/reference standard**	Index test and reference standard
**Number of participants**	Number of participants with and without target condition
**Estimates of accuracy**	Estimates of diagnostic accuracy and their precision (CI)
**General interpretation**	General interpretation of results
**Implication for practice**	Implications for practice
Registration number*	Registration number and name of registry

*Registration number was not coded for in the current investigation

### Additional quality criteria

In addition to the STARD criteria, we also analyzed whether the studies used a validation set to evaluate the performance of the developed methods, which is important because especially deep neural networks pose the danger of overfitting to the training data. Given enough time and a sufficiently large network, all mappings between input and output can be memorized, which can result in perfect performance on the training data. However, such an overfitted network will perform poorly on new data not encountered during training. Therefore, to gain a more realistic assessment of a network’s performance, it is important to test the network on data not encountered during training. Whether a validation set was used in a given study is therefore an important quality indicator to decide whether the performance of a network will generalize. For the purpose of this study, we did not differentiate between internal and external validation. Each abstract was analyzed with regard to whether a validation set (internal or external) not used during training was used to assess the performance of the model.

Additionally, we also analyzed the sample size of each study (i.e., unique participants—not slices or images) and whether the studies reported confidence intervals. Furthermore, we analyzed whether the studies used a public data set and whether the studies evaluated an existing algorithm (commercial or otherwise) that was not developed as part of the study.

### Coding

All coding was done by an experienced radiologist (D.P.) with 8 years of experience in imaging informatics. Each individual abstract was compared to the STARD and quality criteria, and it was decided whether the information demanded by the different criteria was included in the abstract. Criteria had to be explicitly reported to be counted. For instance, even though in some cases the type of data collection (retrospective vs. prospective) could be inferred from the context, studies were only coded as reporting the type of data collection according to the STARD criteria if the type of data collection was mentioned explicitly in the abstract. In addition, for each abstract, the modality, (e.g., CT), body region (e.g., chest), pathology (e.g., lung cancer), and task (e.g., classification) were recorded. For each abstract, only one modality, body region, pathology, or task was selected. If the modality was missing or could not be inferred from the abstract, it was coded as “not available” (*n* = 3). If more than one body region was analyzed in a study, the abstract was sorted into the category “general” (*n* = 7).

### Similarity analysis

The text of all abstracts was compared in a similarity analysis using the Jaccard coefficient. The Jaccard coefficient is the ratio of the intersection over the union of two sets. A Python (version 3.7) [17] script was developed, taking in the full text of each abstract and splitting it into single words, thus creating a set (unordered collection of unique elements) for each abstract. In the next step, all abstracts were compared and the intersection (unique common words) and union (all unique words) of all possible pairs of abstracts were calculated to determine the Jaccard coefficient for each pair. If the Jaccard coefficient was higher than 0.5, then the two abstracts were considered to be highly similar.

## Results

### General characteristics of abstracts

Out of the 184 abstracts included in our analysis, 127 (69.0%) were oral presentations (106 scientific presentation 57.6%; 21 MyT3 presentations 11.4%). The remaining 57 were electronic posters (31.0%). The three countries with the most accepted abstracts were the People’s Republic of China with 58 abstracts (31.5%), Germany with 26 abstracts (14.1%), and India with 15 abstracts (8.2%), see Table 2.

**Table 2:** Distribution of abstracts from participating countries

Country	Number of abstracts	Percent
People’s Republic of China	58	31.5
Germany	26	14.1
India	15	8.2
USA	12	6.5
Netherlands	11	6.0
Italy	9	4.9
UK	7	3.8
Switzerland	6	3.3
Austria	5	2.7
Taiwan	4	2.2
Japan	3	1.6
Republic of Korea	3	1.6
Spain	3	1.6
Brazil	2	1.1
Canada	2	1.1
France	2	1.1
Greece	2	1.1
Hungary	2	1.1
Israel	2	1.1
Lithuania	2	1.1
Turkey	2	1.1
Belgium	1	0.5
Hong Kong	1	0.5
Poland	1	0.5
Portugal	1	0.5
Russian Federation	1	0.5
Saudi Arabia	1	0.5

### Topics

With 87 abstracts (47.3%), CT was the modality most featured in accepted abstracts, followed by MRI (47; 25.5%) and plain radiography (17; 9.2%), see Table 3.

**Table 3:** Modalities featured in accepted abstracts

Modality	Number of abstracts	Percent
CT	87	47.3
MRI	47	25.5
Plain radiography	17	9.2
Mammography	9	4.9
Ultrasound	8	4.3
Nuclear medicine	6	3.3
Text analysis	4	2.2
Other	3	1.6
Not available	3	1.6

The body regions with the most research focus were the abdomen (43; 23.4%), the chest (42; 22.8%), and the brain (32; 17.4%), see Table 4.

**Table 4:** Body regions featured in accepted abstracts

Body region	Number of abstracts	Percent
Abdomen	43	23.4
Chest	42	22.8
Brain	32	17.4
Breast	14	7.6
Cardiac	9	4.9
General	7	3.8
Head/neck	7	3.8
MSK	7	3.8
Prostate	7	3.8
Vascular	6	3.3
Other	5	2.7
Genitourinary	3	1.6
Spine	2	1.1

All abstracts covered a wide range of pathologies and topics. The pathologies/topics most prominently featured in the accepted abstracts were lung nodules (16; 8.7%), image reconstruction (12; 6.5%), and breast cancer (10; 5.4%). Most of the research in the abstracts used machine learning techniques for classification (108; 58.7%), with 67 (36.4%) using deep learning and 41 (22.3%) using radiomics, followed by segmentation (32; 17.4%) and technical applications (21; 11.4%), see Table 5.

**Table 5:** Use cases of machine learning techniques

Task	Number of abstracts	Percent
Classification	67	36.4
Classification (radiomics)	41	22.3
Segmentation	32	17.4
Technical	21	11.4
Detection	13	7.1
Technical (radiomics)	6	3.3
Other	4	2.2

### STARD for abstract adherence results

**Fig. 1:**
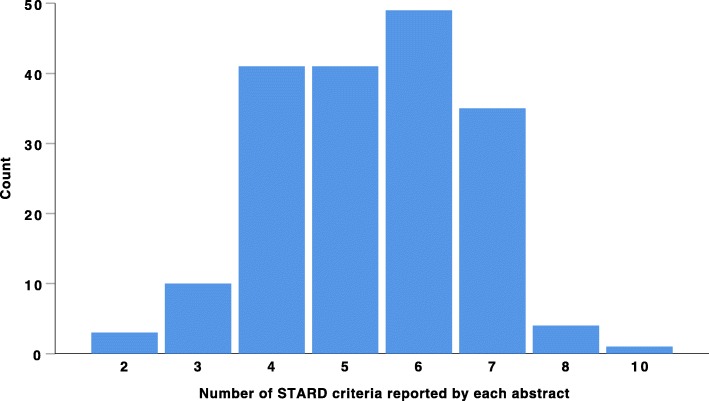
Histogram showing the number of STARD criteria reported by each abstract

Out of the 10 STARD for abstract criteria analyzed in this study, on average, 5.36 (SD = 1.35; Mdn = 5.00) were reported by the 184 abstracts, see Fig. 1. There was no significant difference in the number of reported STARD criteria between abstracts accepted for oral presentation (*M* = 5.35, SD = 1.31) and abstracts accepted for the electronic poster session (*M* = 5.39, SD = 1.45), *t*(182) = − 0.18, *p* = .86, *d* = 0.03.

**Fig. 2:**
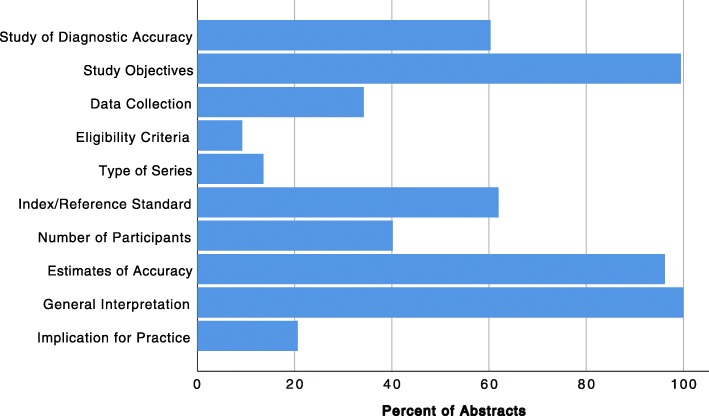
Percent of abstracts that reported individual STARD criteria

One hundred and eleven (60.3%) abstracts stated as their main goal to assess the accuracy of a new method and therefore reported at least one measure of accuracy (sensitivity, specificity, predictive values, or AUC), see Fig. 2. Study objectives were clearly stated by 183 (99.5%) abstracts. With regard to data collection, 63 (34.2%) abstracts reported whether the conducted study was prospective or retrospective. Only 17 (9.2%) abstracts included information about eligibility criteria for participants and settings where the data were collected. Additionally, only 25 (13.6%) abstracts provided information on whether participants formed a consecutive, random, or convenience series. Information about index tests or reference standards was included by 114 (62.0%) abstracts.

Seventy-four (40.3%) abstracts reported the number of participants with and without the target condition that were included in the analyses. The overall majority of abstracts (177; 96.2%) reported estimates of diagnostic accuracy. All abstracts (184; 100.0%) gave a general interpretation of the results. However, only 38 (20.7%) abstracts listed potential implications for practice.

### Additional quality criteria adherence

**Fig. 3:**
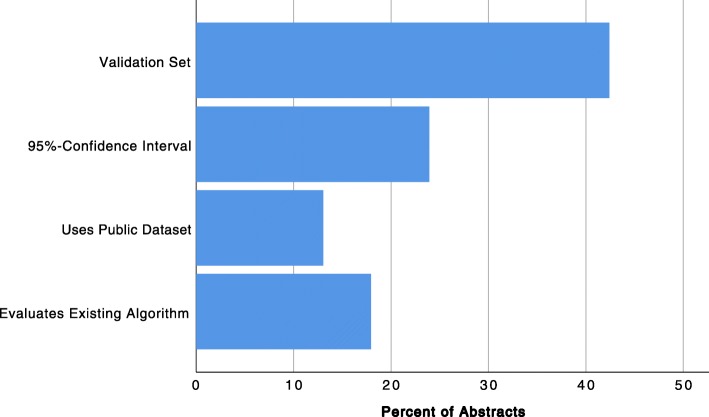
Percent of abstracts that reported additional quality criteria

Seventy-eight (42.4%) abstracts reported having used a validation set for evaluating model performance, see Fig. 3. Confidence intervals were only reported by 44 (23.9%) abstracts. Public data sets to train the models were used by 24 (13.0%) studies, and 33 (17.9%) studies evaluated an existing algorithm (commercial or otherwise) that was not developed as part of the study.

As an additional quality indicator, we also analyzed the number of unique participants per study. One hundred and twenty (65.2%) abstracts reported the unique number of participants. The median sample size was 104, ranging from 7 to 4800.

### Similarity analysis

This analysis returned 3 pairs of abstracts that were highly similar (Jaccard coefficient > .5). Two of those pairs were abstracts in which the same methodology was applied to different pathologies, which resulted in similar terminology being used to sum up the results. However, each study in both pairs constituted an original piece of research. In case of the third pair, the exact same abstract was accepted twice with only minimal changes. Both abstracts were accepted for oral presentation.

## Discussion

Our study of artificial intelligence abstract from ECR 2019 found the following: with regard to international participation, the People’s Republic of China was the country with the most abstracts. The modality with the most research focus was CT. The body regions that were studied the most in the abstracts were the abdomen, the chest, and the brain. All abstracts covered a wide range of pathologies, so no clear focus could be determined. The most prominent use case for machine learning was classification.

With regard to the quality of reporting, the mean number of STARD criteria reported by the abstracts was 5.32. Most papers identified the research as a study of diagnostic accuracy and described the study objectives. Also, a majority of papers cited the index test or reference standard. Some estimates of accuracy were reported by most papers, and a general interpretation of the results was provided. However, there is still room for improvement: details about the data collection, eligibility criteria, and the type of series were rarely reported. Additionally, only few studies provided confidence intervals for the results. And only few studies focused on potential implications for practice. Most notably, only 42.4% of studies used a validation set. As mentioned before, the use of a validation set is crucial when assessing the performance of a given model because overfitting to the test set can lead to artificially inflated performance metrics.

Overall, these results are in line with a previous study on the accuracy of reporting of radiomics in oncologic studies [18]. In that study, 77 articles from high-impact imaging journals were analyzed with regard to adherence to the TRIPOD checklist [19]. Additionally, a Radiomics Quality Score (RQS) taking into consideration demands specific to radiomics studies was calculated for each paper. Similar to our results, papers only reported around half of the information required by the standardized checklist (TRIPOD 57.8%). Furthermore, the mean Radiomics Quality Score, taking into account clinical utility, test-retest analysis, prospective study, and open science, was only 26.1%. These results are not surprising in light of a study on the acceptance of journal reporting guidelines in the field of radiology, which found that only 15% of authors relied on reporting guidelines and checklists when planning their studies [20].

Combining these findings and the results from our own study, it is safe to conclude that the state of reporting of imaging studies using machine learning and radiomics could be improved. One step in this direction could be to move beyond general performance metrics, such as accuracy or area under the curve (AUC), and focus more on diagnostic predictive values, such as positive predictive values (PPV) and negative predictive values (NPV), which may be better suited to estimate whether a given model could be useful for clinical practice [21].

To improve reporting in general, it may be helpful to present authors with a checklist during the submission process, such as STARD (which has been implemented for ECR 2020), so that missing information can be added before submitting the abstract. We think that in several cases, the missing information may have been available but was not added to the abstracts. We are aware of the fact that the word count of the abstracts was limited and authors were not able to add long, detailed descriptions of their studies.

Our study has several limitations: as mentioned before, our study was limited to the quality of reporting. The quality of the studies themselves was not assessed. Therefore, we cannot fully answer the question whether the sharp increase in machine learning-focused publications mainly consists of quality publications with a strong methodology or methodologically flawed studies just following the latest trend.

Based on the current investigation, we cannot determine if there is a correlation between the quality of reporting and the quality of the reported studies themselves. However, a high quality of reporting is the basis for a correct assessment of a scientific study. Thus, future analyses of conference abstracts may include additional quality criteria to further analyze the content and quality of abstracts.

Additionally, even though the topics of the abstracts to the ECR 2019 may give an overview of where the general field of artificial intelligence research in radiology is headed, there are many factors that determine whether an abstract is submitted to a conference. For instance, some authors may have chosen to present their research at different conferences, such as the RSNA. Therefore, abstracts from the ECR 2019 only represent a small part of the research landscape and the trends reflected in the topics should be interpreted carefully. Furthermore, this study only focused on accepted abstracts to ECR 2019. Rejected submissions could not be analyzed.

An additional limitation of the present study is its sole focus on artificial intelligence research. Even though we did find room for improvement with regard to the quality of reporting, this may be true for other areas of research in radiology as well. Thus, future studies should compare the quality of reporting of different areas of radiological research to determine whether compliance with the STARD checklist is particularly low in artificial intelligence research or if compliance is generally low across different disciplines.

In sum, to further improve the quality of abstracts and the review process, we suggest providing both authors and reviewers with simple checklists, such as STARD [13], CONSORT [14], STROBE [15], and PRISMA [16], to ensure that abstracts contain all relevant information and that abstracts are judged based on the same standard. These checklists could be based on the STARD criteria with additional suggestions for machine learning papers. Notably, as studies using machine learning become more frequent, there are efforts to extend existing checklists to include information specific to machine learning research [22, 23]. For instance, a new checklist with 9 key considerations for authors of radiology research on artificial intelligence can serve as a guideline for authors what critical information should be included in a paper or abstract [24]. Regarding double entries, a server-side similarity analysis could be implemented to catch double entries during the submission process.

Taken together, these measures could help to further increase the quality of machine learning research in radiology and improve the communication of scientific results at future conferences.

## Data Availability

The datasets used and/or analyzed during the current study are available from the corresponding author on reasonable request.

## Abbreviations

AUC: Area under the curve
CT: Computed tomography
MRI: Magnetic resonance imaging
STARD: Standards for Reporting of Diagnostic Accuracy Studies
